# Incentives Increase Participation in Mass Dog Rabies Vaccination Clinics and Methods of Coverage Estimation Are Assessed to Be Accurate

**DOI:** 10.1371/journal.pntd.0004221

**Published:** 2015-12-03

**Authors:** Abel B. Minyoo, Melissa Steinmetz, Anna Czupryna, Machunde Bigambo, Imam Mzimbiri, George Powell, Paul Gwakisa, Felix Lankester

**Affiliations:** 1 School of Life Sciences, The Nelson Mandela African Institution of Science and Technology, Arusha, Tanzania; 2 Paul G. Allen School for Global Animal Health, Washington State University, Pullman, Washington, United States of America; 3 Department of Ecology and Evolution, University of Illinois at Chicago. The Lincoln Park Zoo, Chicago, United States of America; 4 Serengeti Health Initiative (SHI), Serengeti, Tanzania; 5 Genome Science Centre and Department of Veterinary Microbiology and Parasitology, Faculty of Veterinary Medicine, Sokoine University of Agriculture, Morogoro, Tanzania; Atlanta Health Associates, Inc., UNITED STATES

## Abstract

In this study we show that incentives (dog collars and owner wristbands) are effective at increasing owner participation in mass dog rabies vaccination clinics and we conclude that household questionnaire surveys and the mark-re-sight (transect survey) method for estimating post-vaccination coverage are accurate when all dogs, including puppies, are included. Incentives were distributed during central-point rabies vaccination clinics in northern Tanzania to quantify their effect on owner participation. In villages where incentives were handed out participation increased, with an average of 34 more dogs being vaccinated. Through economies of scale, this represents a reduction in the cost-per-dog of $0.47. This represents the price-threshold under which the cost of the incentive used must fall to be economically viable. Additionally, vaccination coverage levels were determined in ten villages through the gold-standard village-wide census technique, as well as through two cheaper and quicker methods (randomized household questionnaire and the transect survey). Cost data were also collected. Both non-gold standard methods were found to be accurate when puppies were included in the calculations, although the transect survey and the household questionnaire survey over- and under-estimated the coverage respectively. Given that additional demographic data can be collected through the household questionnaire survey, and that its estimate of coverage is more conservative, we recommend this method. Despite the use of incentives the average vaccination coverage was below the 70% threshold for eliminating rabies. We discuss the reasons and suggest solutions to improve coverage. Given recent international targets to eliminate rabies, this study provides valuable and timely data to help improve mass dog vaccination programs in Africa and elsewhere.

## Author Summary

It is estimated that 59,000 people die from canine-mediated rabies each year, over 99% in developing countries where rabies is endemic and nearly half of the victims are children. The annual global cost has been estimated at 8.6 billion dollars. Yet with highly effective vaccines and a single species of reservoir host (the domestic dog) rabies is entirely preventable through mass dog vaccination. These disease burden statistics, and the evidence that dog vaccination is highly effective at eliminating human rabies, have led the World Health Organisation (WHO), together with the Food and Agriculture Organization (FAO) and the World Organization for Animal Health (OIE), to unite in their joint commitment to the global elimination of canine rabies. To be successful in this, vaccination campaigns must routinely achieve the 70% coverage levels required for rabies elimination. We used a mass dog rabies vaccination campaign in northern Tanzania to assess the accuracy of methods used for estimating coverage. Additionally we assessed the impact that incentives had on vaccination turn out. Our data showed that, despite under- and over-estimating the coverage respectively, both household questionnaire and mark-re-sight surveys were accurate when compared to a gold-standard method for estimating coverage. Given this tendency to provide a conservative estimate, whilst also providing opportunities for valuable demographic data to be collected, we recommend the household questionnaire survey method. Our data also indicated that the provision of incentives did significantly increase the number of dogs brought for vaccination.

## Introduction

Canine rabies has been reported as one of the neglected diseases of the developing world [1,2]. Caused by *Lyssavirus*, it is a zoonotic infection of the central nervous system that invariably leads to death. The disease is transmitted through the saliva of the infected carrier with domestic dogs being the principle infectious source and reservoir of the disease [3]. Endemic in Tanzania, studies show that approximately 1,500 rabies deaths occur annually [4,5].

Many societies develop associations with dogs for different purposes ranging from security, companionship, food acquisition and religious beliefs. Despite these benefits, however, keeping dogs can pose a risk to human health through bite injuries and the transmission of pathogens such as the rabies virus [6].

Although rabies is not considered a human health priority [7], the demand for post-exposure prophylaxis (PEP) in developing countries like Tanzania results in a substantial economic burden due to high costs of vaccination, the direct and indirect costs associated with patient treatment and income loss [8,9]. It is estimated that 3.9 billion people living in in more than 150 countries are at risk from rabies resulting in between 7 and 15 million people receiving PEP and more than 59,000 people dying from rabies each year. Ninety nine percent of these deaths occur in Africa and Asia [9–12]. The annual economic losses associated with rabies have been estimated to be approximately 8.9 billion US dollars [13].

Rabies also impacts the health of wild animal species. In the Serengeti, an ecosystem in northern Tanzania that is surrounded by large agro-pastoralist and pastoralist human populations with relatively dense population of dogs (9.4 dogs/km^2^), repeated outbreaks in the 1980’s and 1990’s dramatically reduced the number of African wild dogs (*Lycaon pictus*) [14,15].

Despite being effective at controlling and or eliminating rabies, vaccination programs targeting domestic dogs are relatively rare in the developing world. Socio-economic factors such as inadequate resources, lack of political commitment, weak inter-sectorial cooperation, limited accessibility to, and the high cost of, modern vaccines, and a general lack of community awareness and cooperation are factors that hamper effective control of rabies in these countries [16–18].

The critical vaccination coverage level of 39% to 57% of the dog population is sufficient to eliminate rabies [19]. However, in areas with high dog population turnover, empirical observations and rabies transmission models suggest 70% of the dog population be vaccinated repeatedly for canine rabies to be eradicated. Indeed, in dog populations with high birth rates and death rates such as in Tanzania, repeat vaccination every few months may be required in order to prevent the herd immunity declining below a critical threshold [4,11].

Since 2003 the Serengeti Health Initiative (SHI) has carried out annual rabies vaccination campaigns in six districts surrounding the Serengeti National Park, typically employing a central-point strategy whereby villages are requested to bring their dogs to a central point for immunization. To monitor coverage following vaccination the proportion of immunized dogs has been estimated using either: i) a randomized household questionnaire survey (HHQ), in which the proportion of vaccinated dogs in a sample of village households is calculated; or ii) a mark-re-sight method (hitherto referred to as transect survey), in which dogs attending the clinic are marked and the proportion of marked dogs estimated by observation during transect surveys on the day following vaccination. These methods are relatively cheap and quick and they have been shown to be feasible in estimating vaccination coverage [3,4,20]. However, to the author’s knowledge, the accuracy of these methods at quantifying rabies vaccination coverage has never been tested.

The vaccination coverage achieved is the critical factor that determines whether the SHI’s campaign to control rabies is successful and it is imperative that the assessment methods used are accurate. The primary objective of this study, therefore, was to measure the accuracy of the two established methods through comparison with a ‘gold-standard’ village-wide census (VWC), whereby every village household was visited and the true vaccination coverage determined.

Vaccination coverage is increased if more villagers bring their dogs to the central point clinic on any given day. A secondary objective, therefore, was to quantify the impact that incentives have on dog owner participation in the central point vaccination clinics.

## Materials and Methods

### Study site

The study was conducted in Bunda and Serengeti (human population 335,060 and 249,420 respectively [21]) Districts of the Mara Region (34°-35°E, 1°30´-2°10´S) in northwestern Tanzania. The districts, which are composed of mixed agro-pastoralist communities, are among the seven districts that SHI has been conducting annual mass dog vaccination campaign since 2003.

### Vaccination clinics

Ten central-point vaccination clinics were carried out in ten villages (*n* = 5 in Bunda District, *n* = 5 in Serengeti District) between June to July and November to December 2013. Vaccination schedule dates were set in advance and, as per the SHI’s standard operating procedure, on the day before vaccination the SHI team visited the targeted village, announcing with loud speakers and posting posters in prominent places that dogs should be brought to the clinic the following day. On arrival at the clinic each dog was registered, and age, sex and prior vaccination history recorded. Following vaccination all dogs were fitted with a brightly coloured collar and marked on both flanks with a stripe of water-soluble purple spray and a vaccination certificate was given to the owner.

### Estimating vaccination coverage

#### Transect survey

Following the completion of a village central point vaccination clinic, three (approximately 3 km) parallel transect routes were established. The primary route ran from the vaccination point with the second and third routes determined based on the location of the first. Since transects were driven by a vehicle, selection of routes was dependent on the suitability of the local roads and were rarely straight. Where possible the primary route passed key landmarks (e.g. the primary school, dispensary, village church or mosque) as passing these points typically enabled the length of the village to be covered. In accordance with the methodology of a previous Tanzanian based study [3], a perpendicular distance of at least 50 m between primary and subsequent routes was maintained in order to minimize the risk of double counting of dogs. The vehicle, with a driver, a recorder and two other observers present, drove along each transect at approximately 15 km/h. Dogs spotted within 20 m of either side of the vehicle were recorded and a note was made of the presence or absence of a collar and / or a paint mark. The start and the end coordinates of each route were recorded (See S1 Table). Each of the three transect routes was driven five times with the first performed on the same day as the vaccination campaign, and the remaining four conducted the following day. All transects were carried out in daylight.

#### Household questionnaire survey (HHQ)

Three days after the end of the vaccination clinic, an HHQ targeting randomly selected households was carried out. A random bearing from the central vaccination point, chosen by spinning a bottle, was walked with every third household selected until the perimeter of the village was reached. As many households as possible were visited in the three-day period (per village) in which the HHQ was carried out, however a minimum of fifteen households per sub-village and 70 per village were selected.

A locally recruited interviewer accompanied the enumerators and verbal consent from the head of the household, or another adult member, was obtained at each house. If no adult was present then the household was not included. The role of the locally recruited interviewer was to facilitate introductions, to request verbal consent and to translate the questions into the vernacular language to ensure understanding. The questionnaire asked how many dogs and puppies (< 6 months of age), human adults and children lived in the household, and the number of dogs and puppies that had been vaccinated at the latest clinic (being confirmed with vaccination cards). If dogs or puppies were not vaccinated the respondents were asked to give the reasons why.

#### Village-wide census (VWC)

The VWC was used to determine the accurate village vaccination coverage. Within a week of vaccination an enumerator together with a well known locally recruited interviewer visited each household in every sub-village to administer, following verbal consent from the head of the household, the survey questionnaire which was the same as the one used in the HHQ. The vaccination coverage results obtained from the VWC were used as a gold standard against which the estimates obtained from the HHQ and the transect survey could be compared.

### Human and dog population characteristics

Demographic data obtained by the HHQ were also used to calculate the proportion of households that keep dogs, the human to dog ratio, and the number of dogs per household and per dog owning household. These demographic data collected by the VWC and the HHQ were compared to determine the accuracy of the latter as a method of demographic data collection.

### Vaccination participation

To evaluate the impact that incentives have on the number of dogs being brought for vaccination (‘turnout’), 62 villages were, in 2013, randomly allocated to four intervention groups: i) vaccinated dogs received brightly coloured collars (*n* = 10), ii) owners that brought a dog were given a brightly coloured wristband (*n* = 8); iii) vaccinated dogs received collars and owners were given a wristband (*n* = 26), and (iv) neither collars nor wristbands were provided (i.e. owners received only vaccination certificates) (*n* = 18). Scheduling challenges arising from the creation and re-designation of new villages in 2013 precluded a more balanced design. In addition data from the SHI’s 2012 vaccination campaign was made available so that the difference in the number of dogs vaccinated in 2012 and 2013 could be calculated. Thereafter we compared the difference in villages that received an incentive in 2013 (village groups i–iii) with the difference in those that received none in both years (village group iv). We also compared turn out in villages that received the different incentive combinations (village groups i–iii).

### Cost of immunization per dog

The costs of immunizing a single dog in a particular village can be calculated by the following equation:

$$cost\;per\;dog=\frac{fixed\;costs+(variable\;costs\;\times \;total\;number\;of\;dogs\;vaccinated)}{total\;number\;of\;dogs\;vaccinated}$$

The fixed costs (salaries, vehicle costs, per diems etc.) were the same in villages with and without incentives given out, whilst the variable costs (syringes, needles, vaccination record cards, plus the cost of the incentive etc.) varied according to how many dogs turn up and whether an incentive was given out.

In villages where incentives were not handed out the variable costs are given by β and in villages where incentives are handed out the variable costs are given by β + γ, where γ is the cost of the incentive. The number of dogs vaccinated is given by *n*:

$$Cost\;per\;dog\;(incentive\;village)\;C1\;=\frac{fixed\;costs+(\beta \;\times \;n)+(\gamma \;\times \;n)}{n_{1}}$$

$$Cost\;per\;dog\;(incentive\;village)\;C1\;=\frac{fixed\;costs}{n_{1}}+\;\beta +\;\gamma$$

$$Cost\;per\;dog\;(non\;incentive\;village)\;C2=\frac{fixed\;costs+(\beta \;\times \;n)}{n_{2}}$$

$$Cost\;per\;dog\;(non\;incentive\;village)\;C2=\frac{fixed\;costs}{n_{2}}+\;\beta$$

For the incentive to be cost effective, therefore:

C1≤C2

$$\frac{fixed\;costs\;(C1)}{n_{1}}+\;\beta +\;\gamma \;\leq \;\frac{fixed\;costs\;(C2)}{n_{2}}+\;\beta$$

To calculate the break-even point we re-arranged the equation to solve for *γ*. In doing so the variable costs drop out of the equation leaving only the fixed costs to determine what the break-even point is and whether the incentive was cost effective:

$$\gamma \;\leq \;\frac{fixed\;costs\;(C1)}{n_{1}}-\;\frac{fixed\;costs\;(C2)}{n_{2}}$$

To parameterize the equation we used the mean fixed cost per village of US$578 [22] and the mean number of dogs vaccinated per village in 2012 (*n*_1_) and the calculated mean when incentives were given out (*n*_2_).

### Cost of vaccination coverage

The costs (labour, fuel etc.) that were incurred while carrying out the VWC, transect survey and household questionnaire were recorded and the average cost per village was calculated.

### Pet owner preference for incentives

Dog owners attending the vaccination clinic in ten villages were asked to rate how likely it would be that they would bring their dogs to the vaccination clinic if they knew that incentives (collars or wristbands) would be given out: very unlikely (1), unlikely (2), no difference (3), likely (4) and very likely (5). In addition, they were asked whether they preferred wristbands or collars.

### Does sub-village distance to the central point affect coverage?

To determine how vaccination coverage at the level of the sub-village is affected by the distance (km) villagers need to walk to reach the central-point clinic, data belonging to the SHI was made available for analysis. The dataset, which was collected by the VWC method, contained coverage data at both the village and sub-village level for ten villages that had been targeted in the 2011 campaign. For each sub-village, the distance in kilometers to the central point and the vaccination coverage, calculated using the same survey questionnaire used in the VWC, are given.

### Data analyses

Data obtained were entered in to spreadsheets using Microsoft Excel 2010 and analyzed using R [23]. An ANOVA of repeated measures, followed by a pairwise *t*-test with Bonferroni adjusted *p*-values, was used to compare the vaccination coverage estimates obtained by HHQ, transect survey and VWC. Paired *t-*tests were used to test the difference in turnout between 2012 and 2103 in villages where incentives were, and were not, given out. Chi-squared and paired t-tests were used to analyse the population characteristic data obtained by the HHQ and VWC. A one-way ANOVA was used to determine the effect that different combinations of incentives had on vaccination turnout. A chi-squared test was used to analyze owner preference for collars and wristbands. A Pearson correlation was used to examine the relationship, at the sub-village level, between distance from the central-point clinic and vaccination coverage.

### Ethical clearance statement

The study was carried out under the supervision of the SHI, which is permitted through the Tanzania Commission for Science and Technology to conduct dog vaccination programs in northern Tanzania (permit number: 2013-275-ER-2005-141). The study was approved by the ethics and research committee of the Nelson Mandela African Institution of Science and Technology Senate and School of Life Sciences and Engineering (permit number: NM-AIST/M.067/T.12). Prior to the administration of the questionnaires verbal consent was obtained from the head of each household or, if not available, an adult family member.

## Results

### Vaccination coverage estimations

**Fig 1:**
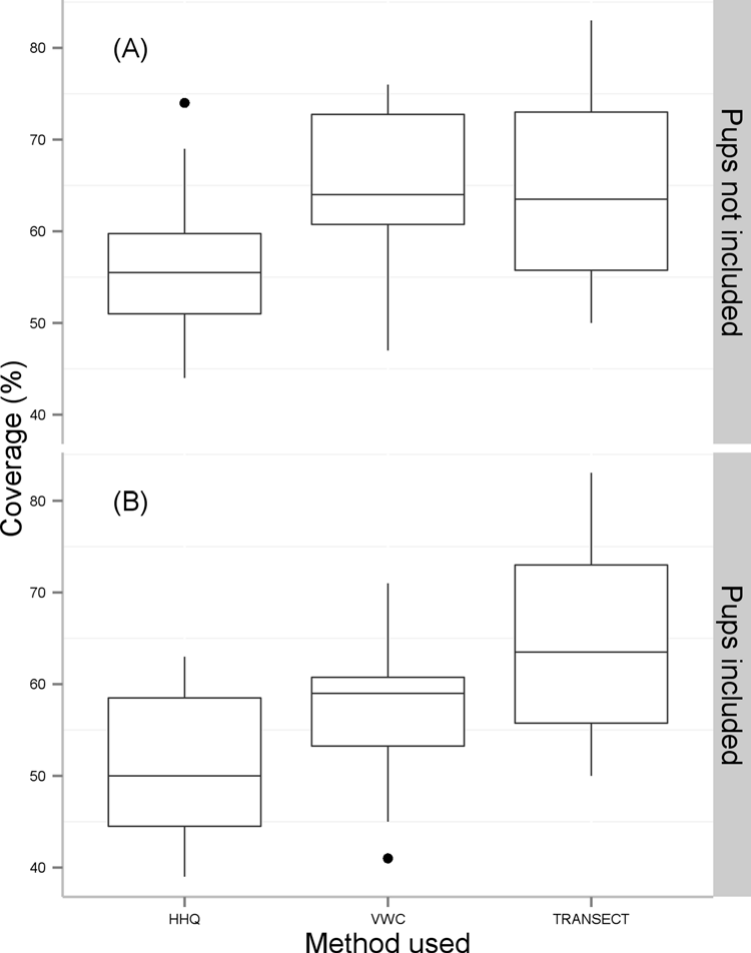
The vaccination coverage as estimated by the different assessment methods (village wide census (VWC), household questionnaire survey (HHQ) and transect survey (TRANSECT). Plot (A) shows coverage when adult dogs and puppies were included in the estimates, and (B) when only adult dogs were included.

The vaccination coverage estimates calculated are shown in Table 1 and Fig 1.

**Table 1:** Vaccination coverage estimated by each of the three comparative assessment methods when puppies were, and were not, included.

Village	Puppies included?	Village-wide census	Household questionnaire	Transect survey
Many'ma	Yes	71	46	74
Tamau	Yes	61	57	61
Ligamba A	Yes	60	63	70
Kiroreli	Yes	41	40	51
Changuge	Yes	45	39	55
Nyibe'kera	Yes	57	60	50
Burunga	Yes	59	47	66
Mbalibali	Yes	59	44	83
Kisangura	Yes	52	53	58
Matare	Yes	69	59	77
Many'ma	No	76	57	74
Tamau	No	63	60	61
Ligamba A	No	73	69	70
Kiroerli	No	47	45	51
Changuge	No	48	44	55
Nyibe'kera	No	74	74	50
Burunga	No	64	54	66
Mbalibali	No	64	51	83
Kisangura	No	60	51	58
Matare	No	72	59	77

### Comparison of coverage estimation methods with adult dogs and puppies included

When both adult dogs and puppies were included, the coverage estimates ranged from 41–71% (mean 57.4%) (VWC), 39–63% (mean 50.8%) (HHQ) and 50–83% (mean 64.5%) (transect survey). The HHQ underestimated the coverage by 6.6%, whilst the transect survey overestimated the coverage by 7.1%. An ANOVA of repeated measures indicated that there was a statistically significant difference between the three tests (df = 2, F = 8.48, *p =* 0.003) with pairwise *t*-tests (with Bonferroni adjusted *p*-values) indicating that the transect survey estimate was significantly different to the estimate of the HHQ (*p =* 0.03). The estimates from the VWC were not different from those of either the HHQ or the transect survey at conventional levels (*p* = 0.14 and 0.06 respectively).

### Comparison of coverage estimation methods with only adult dogs included

When only adult dogs were included, the coverage calculations ranged from 47–76% (mean 64.1%) (VWC), 44–76% (mean 56.4%) (HHQ) and 50–83% (mean 64.5%) (transect survey). The HHQ underestimated the coverage by 7.7% and transect survey overestimated the coverage by 0.4%. An ANOVA of repeated measures indicated that there was no statistically significant difference between the three tests (df = 2, F = 8.48, *p =* 0.06). Pairwise *t*-tests with Bonferroni adjusted *p*-values, however, indicated that the coverage estimate of the HHQ was significantly different to that of the VWC (*p =* 0.01), whilst estimates from the transect survey were not different from those of either the VWC or the HHQ (*p* = 1.0 and 0.34 respectively).

### Cost of vaccination coverage assessment

The VWC was carried out on foot, took two people and, on average, six days to complete. At a daily cost of $20 per worker the average total cost of a VWC was $240 / village. The transect survey consisted of three different 3 km transect routes, taking approximately one hour to complete and driven five times. These activities required four people to work for two days at a cost of $20 / day each, totaling $160. With an approximate fuel consumption of 7 km / litre, and a total of 45 km driven per village, the average fuel use per village was 6.4 litres. With an approximate cost of $1.56 / litre, the fuel cost per village was $10. Therefore the average cost of carrying out a transect survey was $170 per village. The HHQ was conducted by three people and took three days to complete. At a daily cost of $20 per worker, the average cost was $180 per village.

### Impact of incentives on vaccination turnout

**Fig 2:**
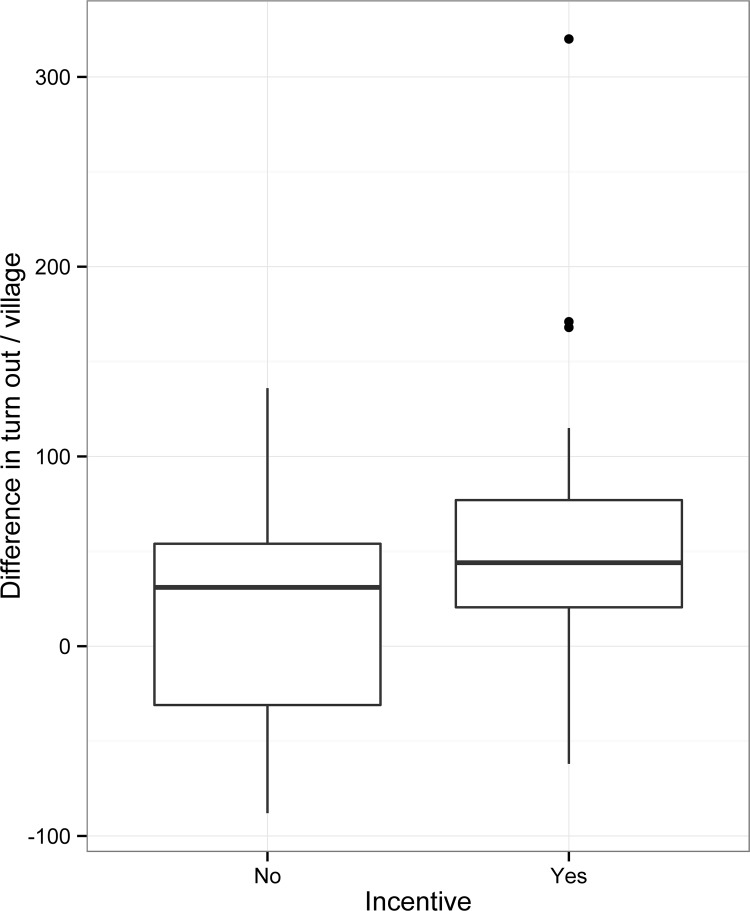
Vaccination turnout. Difference in the number of dogs being brought to vaccination in 2012 and 2013 in villages with and without incentives.

The number of dogs being brought for vaccination in 2012 and 2013 is shown in Table 2. Overall turnout was 23% (mean of 42 dogs / village) higher in 2013 compared to 2012. When turn out in 2012 and 2013 is compared in villages with and without incentives we found that an average of 19 more dogs per village were brought for vaccination in villages without incentives (Fig 2). This increase was not significant (*t* = -1.326, df = 18, *p* = 0.2; 95% CI: -49.2 to 11.1). Whilst on average 53 more dogs were brought in villages with incentives. This was significant (*t* = -5.5187, df = 42, *p* < 0.000001; CI: -72.60324 to -33.72234). Incentives therefore resulted in, on average, 34 more dogs being brought for vaccination per village. Different combinations of incentives had no effect on vaccination turnout (*F* = 0.98, df = 2, 40, *p* = 0.4, 95% CI: 33.7–72.6).

**Table 2:** Vaccination turnout with and without incentives in 2012 and 2013.

Village name	Turnout in 2012	Turnout in 2013	Incentive
Mugeta	549	461	No
Miseke	152	101	No
Rwamchanga	201	139	No
Koreri	222	170	No
Morotonga	280	366	No
Mugumu mjini	998	1134	No
Bwitengi	177	222	No
Bonchugu	249	292	No
Marasomonche	162	157	No
Tabora B	37	98	No
Merenga	207	192	No
Kibeyo	125	183	No
Kebosongo	124	249	No
Robanda	88	73	No
Kitunguruma	154	107	No
Park Nyigoti	147	184	No
Kyambahi	125	175	No
Kegonga	98	123	No
Mbirikiri	90	121	No
Bunda mjini	463	521	Yes
Nyatwali	56	103	Yes
Mariwanda	108	150	Yes
Nyansura	201	521	Yes
Ligamba B	37	73	Yes
Mcharo	69	115	Yes
Kunzugu	39	82	Yes
Changuge	85	168	Yes
Balili	186	357	Yes
Nyamatoke	178	241	Yes
Mihale	188	205	Yes
Sarawe	126	145	Yes
Kiloreli	114	220	Yes
Bukore	99	121	Yes
Hunyari	194	245	Yes
Nyangere	97	65	Yes
Nyamuswa	278	255	Yes
Sarakwa	68	96	Yes
Sanzate	244	299	Yes
Nyaburundu	41	115	Yes
Kurusanga	269	273	Yes
Bunda stoo	302	388	Yes
Ligamba A	131	168	Yes
Tamau	187	301	Yes
Kihumbu	162	157	Yes
Manyamanyama	248	241	Yes
Kitaramaka	147	253	Yes
Salama kati	112	194	Yes
Singisi	197	241	Yes
Nyamisingisi	166	212	Yes
Motukeri	169	190	Yes
Nyakitono	251	271	Yes
Mbalibali	285	260	Yes
Kisangura	140	220	Yes
Iharara	181	193	Yes
Burunga	216	288	Yes
Nyiberekera	311	426	Yes
Kitembere	170	207	Yes
Kono	84	115	Yes
Mbiso	274	314	Yes
Matare	210	378	Yes
Omahe	171	215	Yes
Kenokwe	283	221	Yes

### Cost of immunization per dog

The mean number of dogs vaccinated by the SHI per village in 2012 was 189. With the mean fixed operational cost per village of $578, this results in a cost per dog of $3.06. Use of incentives increased the turn out by an average of 34 dogs per village, resulting in a mean of 223 dogs vaccinated per village at a cost of $2.59 per dog. The break-even cost, under which the incentive must be to be cost-effective, was $0.47.

### Population characteristics

#### Comparing population characteristics determined by the HHQ and VWC surveys

A summary of the data is shown in Table 3. A total of 618 and 2,082 households were visited in the ten study villages during the HHQ and VWC respectively. The HHQ determined that 75% of households kept dogs, whilst the VWC calculated the percentage to be significantly less at 59% (Χ^2^ = 50; df = 1; *p* < 0.0001). The number of dogs per household was closer with 1.8 and 1.87 estimated by the HHQ and VWC respectively (*t* = -0.2613, df = 9, *p* = 0.8). The number of dogs per dog-owning household was the same at 2.6 dogs per household. The human to dog ratio estimated by the HHQ and the VWC were 3.84 and 4.74 respectively. These estimates were not significantly different (*t* = -1.564, df = 9, *p* = 0.15).

**Table 3:** The human and dog population characteristic data collected by either the household questionnaire survey (HHQ) or the village-wide census (VWC). (HH = household; DOHH = Dog owning household; H:D ratio = human to dog ratio.).

Method	District	Village	Dogs / HH	Dogs / DOHH	H:D Ratio
VWC	Bunda	Manyamanyama	1.6	2.7	7.65
VWC	Bunda	Tamau	2.8	3.1	4.03
VWC	Bunda	Ligamba A	1	2.1	6.85
VWC	Bunda	Kiroreli	2.1	2.7	5.43
VWC	Bunda	Changuge	2.5	3.2	3.44
HHQ	Bunda	Manyamanyama	0.8	2.3	4.31
HHQ	Bunda	Ligamba A	0.9	2.4	6.36
HHQ	Bunda	Kiroreli	1.2	2.3	3.69
HHQ	Bunda	Tamau	1.9	2.5	2.71
HHQ	Bunda	Changuge	2.2	3.2	3.25
VWC	Serengeti	Nyibe'kera	2.1	2.6	3.3
VWC	Serengeti	Burunga	1.5	2.5	3.7
VWC	Serengeti	Mbalibali	2	2.4	4.6
VWC	Serengeti	Kisangura	1.5	2.3	4
VWC	Serengeti	Matare	1.6	2.4	4.4
HHQ	Serengeti	Nyibe'kera	1.9	2.6	3.8
HHQ	Serengeti	Burunga	2.7	3	2.4
HHQ	Serengeti	Mbalibali	2.4	2.8	3.3
HHQ	Serengeti	Kisangura	2.1	2.4	4.3
HHQ	Serengeti	Matare	1.9	2.4	4.3

### Collars and wristbands preference

Out of 261 respondents, 107 (41%) preferred dog collars, whilst 113 (43%) preferred wristbands and 41 (16%) had no preference. Approximately 98% of the respondents liked the incentives and were happy to see vaccinated and unvaccinated dogs being easily distinguished.

### Reasons for non-participation in the vaccination campaign

In total there were 738 respondents from the two districts who did not vaccinate some or all of their dogs. The reasons given are as shown in Table 4.

**Table 4:** The frequency and percentage of reasons given by dog owners for non-participation in the vaccination campaigns.

Reason for not vaccinating dogs	Number	Percentage
Dog run away	292	39.6
Didn't hear about vaccination	203	27.5
People not around	102	13.8
Puppies too young	89	12.1
Difficult to handle dog	29	3.9
Late to vaccination point	13	1.8
Don't have time	6	0.8
Dog recently gave birth	4	0.5

### Does sub-village distance to the central point affect coverage?

The data set made available by the SHI is available in the Supplementary Information S1 and contains the sub-village names, their distance from the respective central-point vaccination clinics and the vaccination coverage levels as calculated by a VWC. A Pearson distance–coverage correlation indicated a significant negative relationship between distance and coverage (*r* = -0.27, *t* = -2.1, df = 57, *p* = 0.04) suggesting that as distance to the central-point clinic increased so vaccination coverage at the sub-village level decreased.

## Discussion

This paper provides the first assessment of the accuracy of vaccination coverage estimates made by household questionnaire (HHQ) and transect surveys through comparison with a village-wide census (VWC) “gold standard” method. Additionally, though collars have been commonly used to aid the identification of dogs whilst estimating vaccination coverage [3,20,24], this is the first study to quantify the impact that collars and wristbands used as incentives have on owner participation in mass dog vaccination campaigns.

Two principle findings emerged: i) both of the trial methods, HHQ and transect survey, accurately estimated the vaccination coverage (as compared to the gold standard method of VWC), however the HHQ was significantly less accurate when puppies were not included; ii) there was a significant increase in the number of dogs brought for vaccination in villages where incentives were used.

Our study included the use of wristbands and collars as incentives for the purpose of encouraging community members to bring their dogs for vaccination. We found that the incentives had an impact, increasing vaccination turnout in comparison with villages where no incentives were handed out. Because the villagers were not aware in advance that the collars or wristbands would be handed out, it seems likely that the brightly coloured incentives exerted their effect (when worn by owners or vaccinated dogs) by attracting further owners to bring their dogs to the vaccination points. Logically, and through an economy of scale, the more dogs that turn up for a village vaccination clinic the cheaper the immunization per dog becomes. We calculated the reduction in the cost per dog to be $0.47, which gives a threshold under which the price of the incentive must remain to be cost-effective.

Different combinations of incentives (collars alone, wristbands alone or collars in combination with wristbands) had no significant effect on increasing vaccination turnout. When the owners were asked whether they liked the collars and wristbands, nearly all responded positively and agreed that it was helpful to be able to distinguish vaccinated dogs. As there was no preference for one type of incentive, it would be sensible to invest in dog collars rather than wristbands, as collars can be used both as an incentive and for marking vaccinated dogs.

Mass dog vaccination is the most effective method to control rabies in endemic regions [11]. It is important, however, that 70% of the dog population is immunized to create sufficient herd immunity so that the transmission of the virus is blocked [11,19]. It is important therefore to be able to reliably assess post-vaccination coverage. Carrying out a post-vaccination VWC is a highly accurate method, as data is collected from every household in the village. However, this method is very time consuming and expensive. Quicker and cheaper methods, such as the HHQ and transect survey assessed in this study, are often used [3,4,20]. To the author’s knowledge this is the first time these methods have been validated against a VWC.

Although the HHQ and the transect survey tended to under- and over-estimate the coverage respectively, we found, when puppies were included in the calculation, no significant differences with the vaccination coverage estimates made by the gold standard. However the accuracy of the transect survey estimate was only just above conventional significance. As only adult dogs tend to be visible on a transect survey, and many puppies are not brought for vaccination, this was expected. It follows, therefore, that when puppies were not included in the calculation the transect survey became highly accurate. However the HHQ became significantly less accurate when puppies were not included, underestimating the coverage by an average of 10%. Indeed, whether puppies were, or were not, included the HHQ tended to underestimate the vaccination coverage.

Regarding cost, the transect survey and the HHQ were similarly expensive whilst the VWC was approximately 37% more expensive than both. However the transect survey took less time, and was more simple to carry out. Transect surveys are limited, however, as they only enable vaccination coverage estimation, whereas the HHQ can be modified to include a range of useful demographic data.

In summary, the transect survey compared well with the gold standard whether or not puppies were included, whilst the HHQ was only accurate when puppies were included. Given that within a few months puppies will grow up to become active members of the dog community, it seems sensible to include them in the calculation of vaccination coverage. In this situation one needs to be cautious about relying on an estimate made by a transect survey as we have calculated it to be approximately 7% higher than the real coverage. The HHQ, which tended to underestimate the coverage, provides a more conservative estimate. Given this, the similar cost implications and the potential added value that can be provided by the collection of wider demographic data, we recommend the HHQ. If time is a constraint, however, the transect survey provides a quicker method of assessment.

Despite the positive impact that incentives had on vaccination turnout, the vaccination coverage estimates from the ten villages in our study were on average 16% below the recommended coverage of 70% required to disrupt rabies [11]. The reasons given for non-participation in the clinics were consistent with other studies [20,24], with over a third of respondents who had not brought their dogs for vaccination claiming their dogs had run away, whilst others thought their puppies were too small for vaccination. Given the impact that unvaccinated puppies have on vaccination coverage, this reason should be addressed by sensitizing owners to the fact that puppies of any age can be effectively immunized [25] and that failure to vaccinate them may allow rabies to persist [4,11,18]. Despite the advertising campaign carried out the day before the clinic, over a quarter of the respondents that did not bring their dogs claimed to have been unaware of the vaccination campaign. This could be attributed to the difficulty in accessing all areas of a village when advertising the campaign by loudspeaker from a vehicle. Although likely to take considerably longer, advertising by foot, so that all sub-village areas are targeted, will probably improve this.

The vaccination campaign in the study villages was carried out during the season of cultivation and it is likely that a proportion of the respondents that said they were not at home were busy with farming activities which prevented them attending vaccination. Scheduling campaigns to take place outside of harvesting or planting season might help to further increase coverage. Additionally, having been the target of rabies vaccination campaigns for over ten years, it is possible that the reduced incidence of rabies in the target villages has caused people to become complacent about the disease; a pattern of behaviour that can lead to the re-establishment of rabies. Furthermore, Tanzanian villages are typically large with widespread sub-village areas, requiring people living at the periphery to travel long distances on foot to reach a central-point vaccination clinic. Although having to walk a long way to the central-point was not given as a reason for non-attendance the findings from the sub-village distance–coverage correlation indicated that vaccination coverage, at the level of the sub-village, decreases as the distance to the central point increases. Carrying out secondary satellite clinics in peripheral sub-village areas, or visiting remote households on foot, would address this but would also increase costs considerably.

The collection of demographic data allows the human and domestic dog population characteristics to be characterised, which is important for the design of vaccination campaigns. Although the HHQ significantly overestimated the proportion of dog owning households, the other estimates, including the human to dog ratio, important for planning vaccination campaigns, compared well with the gold-standard data collected by the VWC. Further, the human to dog ratios estimated were consistent with previous studies in rural areas [24,26,27], but were considerably lower when compared to urban and rural coastal areas [3,6]. As pet ownership in urban and coastal areas, particularly Muslim coastal communities, has been shown to be less popular [6] this finding was not surprising.

In conclusion, we find that vaccination coverage estimates determined by both household questionnaire and transect surveys were accurate when all dogs were included in the calculations, and that both methods can be relatively cheaply employed. However as the transect survey tends to overestimate the coverage, caution is required when using this method. Given the added value of the accurate demographic data that can be obtained through HHQ, and that this method tends to provide caution by underestimating coverage, we conclude this method to be preferable. The use of collars and wristbands as incentives in dog vaccination coverage clinics had a significant impact on vaccination turnout and, assuming cheap options are available, they can reduce the cost of immunization per dog and we therefore recommend their use. However improving the dissemination of advertising, for example by involving local leaders to ensure that the importance of participation, by dogs of all ages, is transmitted to the most remote areas of each village, is required. Furthermore scheduling campaigns around key farming activities might further improve coverage.

## Supporting Information

S1 Table Sub-village names, their distance from the respective central-point vaccination clinics and the vaccination coverage levels obtained. (XLS)
